# Evaluation of a Diabetes Prevention Intervention for Korean American immigrants at Risk for Diabetes

**DOI:** 10.1089/heq.2021.0137

**Published:** 2022-03-03

**Authors:** Simona C. Kwon, Laura C. Wyatt, Susan S. Kum, Jennifer M. Zanowiak, Sara S. Kim, Stella S. Yi, Deborah Min, Linda Lee, Nadia S. Islam

**Affiliations:** ^1^ Department of Population Health, NYU Grossman School of Medicine, New York, New York, USA.; ^2^ Evernorth, St. Louis, Missouri, USA.; ^3^ Korean Community Services of Metropolitan New York, New York, New York, USA.

**Keywords:** randomized controlled trial, diabetes education, Asian Americans, Korean Americans, lifestyle intervention

## Abstract

**Purpose:**

Despite the small but growing number of studies documenting the increasing prevalence of diabetes among Korean Americans, no culturally adapted interventions have been developed for Korean Americans at risk for diabetes. We evaluate the efficacy of a culturally tailored lifestyle intervention among Korean American immigrants at risk for diabetes in New York City (NYC).

**Methods:**

Korean Americans at risk for diabetes were recruited into a culturally adapted, community health worker (CHW) intervention in NYC. Treatment group participants received 6 group sessions and 10 follow-up phone calls from CHWs over the 6-month period. Control participants received only the first session. Study outcomes included changes in weight, body mass index (BMI), blood pressure, physical activity (PA) and PA behaviors, nutrition behaviors, and diabetes knowledge. Paired *t*-tests and chi-square tests assessed group differences for each group for each outcome measure.

**Results:**

The treatment group reported significant positive changes in recommended weekly PA, PA self-efficacy, PA barriers, nutrition self-efficacy, diabetes knowledge, weight, BMI, and systolic blood pressure compared with control participants. Generalized estimated equations models for repeated measures assessed change across time while adjusting for study arm, time point, and the interaction between study arm and time point. The intervention effect was significant for weekly moderate and vigorous PA, recommended weekly PA, PA self-efficacy, and diabetes knowledge.

**Conclusions:**

Results suggest that a culturally adapted lifestyle intervention for Korean American immigrants at risk for diabetes have the potential to improve behaviors associated with cardiovascular disease outcomes and diabetes prevention. Further research among Korean Americans is warranted.

## Introduction

Type 2 diabetes affects ∼30.3 million people, or 9.4% of the U.S. population.^1^ The prevalence of type 2 diabetes is higher among racial/ethnic minority groups such as Blacks and Hispanics compared with non-Hispanic Whites, but many Asian Americans also have a higher prevalence. For instance, 2017–2018 National Health Interview Survey data found that Asian Americans had a diabetes prevalence of 9.2%, compared with 7.5% among non-Hispanic Whites.^1^ Similarly, 2013–2016 National Health and Nutrition Examination Survey data found a higher diabetes and prediabetes prevalence among Asian Americans compared with non-Hispanic Whites (11.2% vs. 9.4% and 33.0% vs. 31.0%, respectively).^1^

Owing to aggregation of Asian subgroups in collection, reporting, and analysis of data,^2–4^ there is limited research on diabetes among Korean Americans. A number of studies have looked at disaggregated electronic health record data from large health care organizations in California; although diabetes prevalence among Koreans in these studies is often lower than other Asian American subgroups, the prevalence is generally higher than among non-Hispanic Whites.^5–8^

A study in New York City (NYC) utilizing survey data from 2009 to 2011 found diabetes prevalence among Korean Americans to be 10.0%, whereas a 2012–2013 community screening in California found a diabetes prevalence of 11.9% among Korean Americans.^9,10^ According to the California Health Interview Survey, the diabetes rate among Korean Americans in California has increased from 4.8% in 2003 to 11.3% in 2017.^11^ Data from the Health Atlas found that diabetes prevalence among Korean Americans in Los Angeles County was 14%, whereas the prevalence in NYC was 7% (NYC Community Health Survey) and 12% (NYU Community Health Needs & Needs Assessment data).^12^

The National Diabetes Prevention Program (DPP) was created in 2010 to address the increasing burden of type 2 diabetes and prediabetes in the United States. It includes a Centers for Disease Control and Prevention (CDC)-recognized lifestyle change program, with a focus on healthy eating and physical activity (PA).^13,14^ DPP demonstrated that diet and lifestyle counseling was more effective than metformin in reducing the incidence of diabetes.^15^ DPP has been culturally adapted to engage diverse populations, and a subanalyses across racial and ethnic groups found that the average weight loss was consistent in the 5–7% range.^16^

Cultural adaptation has been defined as “the systematic modification of an evidence-based treatment or intervention protocol to consider language, culture, and context in such a way that is compatible with the [community's] cultural patterns, meanings, and values.”^17^ A key component of cultural adaptation is the integration of both observable and cognitive aspects of a local culture into intervention content.^18^ Examples include identifying health workers of the same background or culture, considering appropriate cultural concepts and metaphors when developing translations, and incorporating nutrition information that reflects foods and recipes familiar to a community. Evidence-based lifestyle interventions must be implemented in community settings in a way that is culturally sensitive and sustainable to be effective.^19–22^

Lifestyle factors such as unhealthy eating habits and inadequate PA are risk factors of diabetes.^23^ As a whole, Asian Americans engage in lower levels of leisure-time PA as compared with other minority groups.^24–26^ In addition, Korean Americans report very low rates of PA and rank PA as the lowest priority among health promotion behaviors.^27–29^

Similarly, the eating habits of more acculturated Korean Americans may be associated with cardiovascular disease (CVD) risk factors, including diabetes and hypertension. A study utilizing 2011–2016 National Health and Nutrition Examination Survey data found that more acculturated Asian American adults had lower scores for total vegetables and dietary fibers, and higher intakes of added sugars and fats, as well as higher body mass index (BMI).^30^

A study examining the eating habits of Korean Americans in the Midwest found that participants had a higher intake of salt and calories, but a low consumption of dairy, compared with other immigrant populations.^31^ In the NY metropolitan area, a study found that consumption of sweets was significantly related to the level of acculturation, with higher acculturation related to a greater consumption of foods in the sweet group.^32^

In Korea, where dietary patterns have been changing to resemble more western style dietary patterns, a study investigated the use of Korean traditional diets to improve HbA1c and blood pressure (BP) among diabetic and hypertensive patients; those utilizing traditional foods had greater changes than those eating usual foods.^33^ It should also be noted that high sodium intake is associated with high BP and obesity through high energy intake; traditional Korean diets are high in sodium (e.g., soy sauce, pickled vegetables, and bean paste), and combined with a western diet (e.g., animal protein, refined sugar, and fats) may increase the risk for CVD.^34,35^

Despite the effectiveness of DPP programs across racial and ethnic communities in the United States, there have been no DPP-adapted studies specific to Korean Americans. The objective of this study was to evaluate a culturally adapted, community-based intervention informed by DPP to promote diabetes prevention and healthy lifestyle behaviors among Korean Americans in NYC identified as at risk for diabetes. Specifically, we examine changes in PA, nutrition, diabetes knowledge, weight, BMI, and BP.

## Methods

### Study design and conceptual framework

Project RICE (Reaching Immigrants through Community Empowerment) was a two-arm randomized controlled trial (RCT) designed to promote diabetes prevention and healthy lifestyle behaviors in a NYC Korean American population conducted between May 2011 and September 2014.

All aspects of the project were guided by the principles of community-based participatory research (CBPR), and involved a coalition of community partners, researchers, health providers, and community health workers (CHWs).^36^ The university worked in partnership with a community service organization serving Korean Americans in the metropolitan NYC area on cultural adaptation and implementation of the project, research analysis, and dissemination of findings. The current study builds upon previously published pilot data.^37^

### Study population

Inclusion criteria included the following: (1) self-identification as Korean American; (2) 18–75 years of age; and (3) at risk for diabetes using a diabetes risk assessment tool adapted from the American Diabetes Association (based on age, gender, gestational diabetes diagnosis, family history of diabetes, high BP diagnosis, BMI, and prediabetes diagnosis).^38^ Ineligibility included: (1) confirmed diabetes from a health professional; (2) a serious health problem (e.g., terminal illness); (3) participation in a previous CVD study; (4) planned travel for longer than 6 weeks during the intervention period; or (5) pregnancy. The study protocol was approved by the NYU School of Medicine Institutional Review Board and the trial was registered at ClinicalTrials.gov (NCT03530579).

### Screening and randomization

**FIG. 1.**
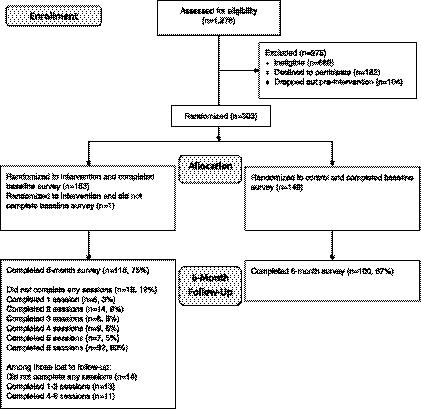
CONSORT diagram for the RICE project. RICE, Reaching Immigrants through Community Empowerment.

Study recruitment was conducted in community centers, faith-based organizations, and cultural events in the borough of Queens. After consenting to participate, individuals completed a screening survey. A total of 1,278 potential participants were assessed for eligibility. Of the 302 participants who consented and completed the baseline assessment, 153 were randomized to the intervention group and 149 were randomized to the control group. Randomization was performed by gender. Husband and wife pairs were randomized into the same group, using the randomization status of the wife. Randomization occurred after the first protocolized health education session and completion of baseline survey assessment (Fig. 1).

### Intervention

The 6-month intervention delivered in Korean by four trained, bilingual Korean American CHWs was based at the community organization and supported by additional programmatic staff from the organization. Session topics were culturally adapted from the DPP curricula and translated into Korean using an iterative and collaborative process between the community–academic partnership and piloted before study implementation.

A total of six sessions were facilitated by the CHWs in a convenient community setting and were ∼2 h in length. Attendance varied by session and round, but was typically between 3 and 10 individuals, with the largest number being ∼20. Session topics included: (1) an overview of diabetes prevention; (2) nutrition; (3) PA; (4) diabetes complications and other CVDs; (5) stress and family support; and (6) access to health care.^14^

Culturally adapted components included the following: discussion of diabetes prevalence and risk in Asian American communities and dispelling common cultural misconceptions regarding diabetes; healthy adaptations using traditional Korean foods and recipes; and integrating family-centered and intergenerational messaging. These components are further detailed in the pilot paper.^37^ The project curriculum incorporated materials from the National Heart, Lung, and Blood Institute's Healthy Heart, Healthy Family; the DPP; and the National Diabetes Education Program's Power to Prevent and Road to Health curricula.^13,39^

Treatment group participants received 10 bi-monthly follow-up phone calls from a CHW during months 1–5, lasting from 15 to 25 min depending on the challenges or needs of the participant. During each call, CHWs reinforced session learning content and discussed individualized challenges, strategies, and goal-setting activities using motivational interviewing. Participants were asked to develop nutrition or exercise-related goals in which to maintain a healthy weight (e.g., going for a walk or reducing fat intake). The CHW recorded the information in the call, as well as additional information on how the participant was doing with their health goal, potential strategies, and follow-ups on referral or health needs. The goal setting support was guided by the Health Belief Model and social support theory.

Control group participants received only the first educational session.

### Outcome and measures

Treatment and control participants completed a survey at baseline, and follow-up surveys were administered at 3 and 6 months. All survey questions were developed in English and translated into Korean by study staff at the community partner organization. Demographics were captured at baseline, and all other measures were captured at baseline, 3, and 6 months. Measures are given in Table 1.

**Table 1.** Questions Asked for Physical Activity, Nutrition, and Diabetes Knowledge

Weekly PA	
Including what you do at your job, home, gym, or elsewhere, do you do any sustained PA for 10 min or more?	Yes No
During the last 7 days, on how many days did you do moderate physical activities?	(Days per week)
What moderate physical activities did you perform?	Brisk walking Carrying shopping bags or laundry Gardening Stretching Other (Specify)
How much time did you usually spend doing these moderate types of physical activities on a normal day that you do activity?	(Minutes per day)
During the last 7 days, on how many days did you do activities that required large amounts of physical exertion or effort to make your heart rate and breathing much faster?	(Days per week)
What large effort physical activities did you perform?	Running or jogging Lifting weights or heavy loads Aerobics Other (Specify)
On one of those days, how much time did you usually spend doing these hard types of physical activities?	(Minutes per day)
PA self-efficacy	
How sure you do feel that you will be able to know what exercises are healthy for you?	Not at all sure Not very sure Somewhat sure Very sure
How sure do you feel that you will be able to exercise for at least 30 min five times each week in the future?	
PA barriers	
I don't have enough time to exercise	Agree Disagree
I am not motivated to exercise	
I don't have a safe place to exercise	
Health problems keep me from exercising	
I don't like to exercise	
I need someone to exercise with but don't have one	
I don't know what exercises to perform	
PA social interaction	
How often do you suggest doing something active when you get together with family members or friends, such as going for a walk, biking, or swimming?	Almost never Sometimes Often Almost always
How often do you set aside a special time to do PA?	
How often do you ask a friend or relative to do some PA with you?	
How often do you talk to others about the benefit of PA?	
Portion control	
How often do you stop eating when full?	Almost never Sometimes Often Almost always
How often do you refuse offers of food when you are not hungry?	
How often do you try to limit the number of food servings you ate?	
How often do you try to limit the size of food servings you ate?	
How often do you try to find something else to do instead of snacking?	
Barriers to healthy eating	
It is difficult for me to choose a healthy snack	Agree Disagree
I cannot afford to buy healthier foods	
I do not have the time to prepare healthier foods	
There is no store for me to buy healthy foods	
It is difficult for me to eat healthy foods on holidays or on special occasions	
It is uncomfortable for me to refuse unhealthy foods when they are offered to me at get-togethers	
I do not like how healthier foods tastes	
I do not cook healthy foods because my family does not like them	
Nutrition self-efficacy	
Are you confident that you can stay on a healthy diet?	Yes No
Are you confident that you can cook a healthy diet?	
Are you confident that you can decrease the amount of sugar and sweets you eat?	
Are you confident that you know what foods you should eat on a healthy diet?	
Are you confident that you can stay on a healthy diet when eating outside your home?	
Are you confident that you can stay on a healthy diet when you're busy?	
Diabetes knowledge scale 1	
How much does each of the following affect a person's risk for getting diabetes?	Increases or raises the risk Has no effect Decreases or lowers the risk
Being Korean American (increases)	
Eating a healthy diet (decreases)	
Having had diabetes during pregnancy (increases)	
Having a blood relative with diabetes (increases)	
Being 65 years of age or older (increases)	
Exercising regularly (decreases)	
Controlling weight gain (decreases)	
Diabetes knowledge scale 2	
Can a person get diabetes if he or she has a normal body weight?	Yes^a^ No
Which of the following is highest in carbohydrate?	Baked chicken Rice^a^ Cheese Peanut butter Don't know
Eating foods lower in fat decreases your risk for…	Nerve disease Kidney disease Heart disease^a^ Eye disease Don't know
Which of the following is not usually associated with diabetes?	Vision problems Kidney problems Nerve problems Lung problems^a^ Don't know
Empty calories is a term used to describe foods which supply calories and no other nutrients. Which of the following are sources of “Empty Calories” (can check more than one)	Fruit juice Margarine^a^ Soft drinks^a^ Sugar^a^ Don't know
Insulin causes blood sugar to:	Decrease^a^ Increase Neither A nor B above Don't know
How much exercise or PA is recommended for most adults to get each week?	90 min each week 10 min every day 15 min for 5 days each week 150 min each week^a^ Don't know

^a^ Correct answer.

PA, physical activity.

#### Weekly PA

A series of questions assessed self-reported moderate and vigorous weekly PA, adapted from the Behavioral Risk Factor Surveillance System.^40^ Total days per week and total minutes per day of average activities were reported. Checkboxes for type of activity were included on the survey to further categorize moderate and vigorous activities correctly. Moderate PA choices included brisk walking, carrying shopping bags or laundry, gardening, and stretching, whereas vigorous PA choices included running or jogging, lifting weights or heavy loads, and aerobics. Additional write-in responses included yoga and golf, categorized as moderate PA, and swimming and biking, categorized as vigorous PA.

Recategorization of groupings was performed when necessary (e.g., moving golf from vigorous to moderate totals). Weekly totals for moderate and vigorous PA were calculated using the following equation: days×min. Based on 2008 PA guidelines, it is recommended that adults perform ≥150 min of weekly moderate-intensity PA or ≥75 min of weekly vigorous-intensity PA.^41,42^ Weekly recommended PA was calculated using the following equation: total minutes of weekly moderate PA + (total minutes of weekly vigorous PA×2). Individuals engaging in ≥150 min per week of PA met weekly recommended PA.^41,42^

#### PA scales

PA self-efficacy was adapted from Bandura's self-efficacy scale,^43^ and included two questions. Reponses included: not at all sure (1), not very sure (2), somewhat sure (3), and very sure (4); the mean was calculated for a scale of 1–4, with 4 representing highest self-efficacy. Cronbach's alpha was 0.539 (baseline) and 0.677 (6 months).

PA barriers were adapted from the Exercise Benefits and Barriers scale^44^ and included seven questions. Responses included agree (1) and disagree (0); question responses were totaled for a scale of 0–7, with 7 representing the greatest barriers to exercise. Cronbach's alpha was 0.621 (baseline) and 0.671 (6 months).

PA social interaction was adapted from a previous intervention on weight management,^45^ and included four questions. Reponses included the following: almost never (1), sometimes (2), often (3), and almost always (4); the mean was calculated for a scale of 1 to 4, with 4 representing highest social interaction. Cronbach's alpha was 0.832 (baseline) and 0.911 (6 months).

#### Nutrition

Portion control questions were adapted from a previous intervention on weight management.^45^ Portion control included five questions; responses included the following: almost never (1), sometimes (2), often (3), and almost always (4); the mean was calculated for a scale of 1–4, with 4 representing the highest portion control. Cronbach's alpha was 0.807 (baseline) and 0.802 (6 months).

Barriers to eating healthy were adapted from previous interventions,^46–48^ and included eight questions. Responses included agree (1) or disagree (0); question responses were totaled for a score of 0–8, with 8 representing the highest barriers. Cronbach's alpha was 0.642 (baseline) and 0.733 (6 months).

Nutrition self-efficacy was adapted from the Bandura Self-Efficacy Scale, and included six questions. Responses included yes (1) or no (0); responses were totaled for a score of 0–6, with 6 representing the highest self-efficacy. Cronbach's alpha was 0.685 (baseline) and 0.679 (6 months).

#### Diabetes knowledge

Two scales assessed diabetes knowledge. The first was developed using the Diabetes Knowledge Test,^38^ and the second scale included questions adapted from the Michigan Diabetes Knowledge Test.^49^ For both, correct was coded to 1 and incorrect was coded to 0; responses were summed for a total score of 0–7, with 7 representing the highest knowledge.

Physiological measures include weight, height, systolic blood pressure (SBP), and diastolic blood pressure (DBP). All measures were collected by CHWs and study staff. Height was collected at baseline using a tape measure taped to the wall. Weight was recorded using a scale, and BMI was calculated using baseline height. BP was measured using an Omron HEM-712C automatic BP monitor. Three resting BP measurements were taken while participants were in a seated position; the second and third measurements were averaged. Glucose and cholesterol were collected by the CHWs at baseline using finger prick.

### Statistical analyses

Descriptive statistics summarize the baseline characteristics of the treatment and control groups; Pearson chi-square tests assess group differences for categorical variables and Student's *t*-tests assess group differences for continuous variables. To assess change between groups for each continuous outcome measure, generalized estimated equations (GEE) models for repeated measures over time were run using PROC GENMOD in SAS while adjusting for study arm, time point (baseline, 3, and 6 months), and the interaction between study arm and time point (the intervention effect), age, gender, education, years lived in the United States, and insurance status.

The interaction variable indicates if there are significant differences in changes in the outcome between the treatment and control groups. Missing data were excluded from analysis unless >5%. To assess change between groups for the dichotomous outcome of recommended PA, a GEE model using a binomial distribution to estimate odds ratio was run. SAS version 9.4 (SAS Institute, Cary, NC) and R 3.5.2 were used for analyses.

## Results

Baseline sociodemographics and outcome measures for the 302 randomized individuals are presented (Table 2). Among the entire group, 58% were women, mean age was 61.2 (standard deviation [SD]=8.0), all individuals were born outside of the United States, mean years in the United States was 22.3 (SD=10.2), 85% were married or living with a partner, and 71% spoke English not well or not at all. Mean BMI (kg/m^2^) was 25.1 (SD=3.5), mean weight (lbs) was 141.9 (SD=22.4), mean SBP was 131.8 (SD=16.7), mean DBP was 81.4 (SD=10.7), mean moderate weekly PA (min) was 142.3 (SD=319.9), mean vigorous weekly PA (min) was 65.2 (SD=165.7), and 46% had met weekly PA recommendations.

**Table 2.** Baseline Characteristics of Individuals Randomized to the Reaching Immigrants through Community Empowerment Project

	***n***	Total (***n***=302)	Treatment (***n***=153)	Control (***n***=149)	***p***
Sociodemographics					
Female, %	302	58.3	58.8	57.7	0.846
Years of age, mean (SD)	299	61.2 (8.0)	62.2 (7.8)	60.2 (8.2)	0.033
Years lived in the United States, mean (SD)	302	22.3 (10.2)	22.5 (10.0)	22.1 (10.5)	0.727
Married/living with partner, %	301	84.7	83.6	85.9	0.571
Education, %	285				0.429
<High school		10.2	11.6	8.6	
High school/some college		49.5	51.4	47.5	
College graduate or higher		40.3	37.0	43.9	
English proficiency, %	299				0.560
Very well/well		28.8	30.3	27.2	
Not well/not at all		71.2	69.7	72.8	
Employed, %	299	51.2	48.0	54.4	0.271
Insurance, %	300				0.465
Private		39.3	42.8	35.8	
Public		12.3	11.8	12.8	
Uninsured		48.4	45.4	51.4	
PA, mean (SD)					
Weekly moderate PA, min	295	142.3 (319.9)	121.3 (177.0)	164.6 (421.4)	0.257
Weekly vigorous PA, min	296	65.2 (165.7)	49.9 (124.9)	81.4 (199.2)	0.106
Recommended weekly PA, %	295	46.4	44.7	48.3	0.545
Self-efficacy, 1–4, 4=highest	297	3.0 (0.7)	3.0 (0.6)	3.0 (0.7)	0.965
Barriers, 0–7, 7=most	301	1.5 (1.6)	1.5 (1.6)	1.5 (1.6)	0.841
Social interaction, 1–4, 4=highest	302	2.1 (0.7)	2.0 (0.7)	2.1 (0.8)	0.480
Nutrition, mean (SD)					
Portion control, 1–4, 4=highest	284	2.9 (0.8)	2.9 (0.8)	2.8 (0.8)	0.271
Barriers, 0–8, 8=most	301	4.9 (2.1)	4.8 (2.2)	5.0 (2.0)	0.498
Self-efficacy, 0–8, 8=highest	302	5.0 (1.3)	5.1 (1.3)	5.0 (1.3)	0.207
Diabetes knowledge, mean (SD)					
Scale 1, 0–7, 7=highest	302	5.5 (1.4)	5.5 (1.5)	5.5 (1.4)	0.780
Scale 2, 0–7, 7=highest	302	3.9 (1.3)	3.8 (1.3)	4.0 (1.2)	0.160
Physiological measures					
BMI (kg/m^2^)	302	25.1 (3.5)	25.5 (3.7)	24.7 (3.1)	0.046
Weight (lbs.)	302	141.9 (22.4)	142.6 (21.4)	141.2 (23.5)	0.569
SBP (mm Hg)	296	131.8 (16.7)	131.6 (15.8)	132.1 (17.6)	0.788
DBP (mm Hg)	296	81.4 (10.7)	81.4 (10.9)	81.5 (10.6)	0.963
Glucose	297	109.3 (28.7)	107.9 (21.5)	110.8 (34.6)	0.397
Cholesterol, mean (SD)	297	203.7 (53.3)	202.0 (49.9)	205.5 (56.6)	0.575

BMI, body mass index; DBP, diastolic blood pressure; SBP, systolic blood pressure; SD, standard deviation.

Additional scales and outcome measures at baseline are presented (Table 2). At baseline, the treatment group had a significantly higher mean age compared with the control group (62.2 vs. 60.2, *p*=0.33) and the treatment group had significantly higher mean BMI compared with the control group (25.5 vs. 24.7, *p*=0.046). We also examined differences in sociodemographic baseline measures between individuals completing follow-up and not completing follow-up by treatment and control groups; no significant differences were found.

Among treatment group participants, follow-up phone calls ranged from 0 to 10, with ∼13% not receiving any follow-up phone calls, and 53% receiving at least 8. Mean calls was 6.7 (SD=3.7). Total sessions ranged from 0 to 6, with 60% completing all 6 sessions, 71% completing at least 4, and 12% completing no sessions.

In the treatment group, significant positive changes were seen for weekly moderate PA, weekly vigorous PA, recommended weekly PA, PA self-efficacy, PA barriers, nutrition self-efficacy, both diabetes knowledge scales, weight, BMI, and DBP between baseline and follow-up; in the control group, significant positive changes were seen for nutrition barriers, nutrition self-efficacy, and the second diabetes knowledge scale (Table 3). The control group also saw a significant decrease in weekly vigorous PA, as well as nonsignificant decreases in moderate PA, recommended weekly PA, PA self-efficacy, and PA social interaction.

**Table 3.** Changes Over 6 Months and Final Adjusted Generalized Estimated Equations Models, Reaching Immigrants through Community Empowerment Project

	Treatment group, mean (SD)				Control group, mean (SD)				Intervention effect or OR	
	***n***	Baseline	6 months	***p***	***n***	Baseline	6 months	***p***-	Adjusted^a^	***p***
PA										
Weekly moderate PA, min	114	115.9 (167.1)	162.4 (187.0)	0.012	94	199.6 (508.7)	120.5 (155.6)	0.140	48.1 (4.1, 92.0)	0.032
Weekly vigorous PA, min	113	57.5 (133.3)	119.2 (171.8)	<0.001	97	98.7 (231.2)	60.4 (125.0)	0.029	41.2 (20.3, 62.0)	<0.001
Recommended weekly PA, *n* (%)	114	54 (47.4)	71 (62.3)	0.025	94	49 (52.1)	45 (47.9)	0.564	1.5 (1.0, 2.1)	0.048
Self-efficacy	112	3.0 (0.7)	3.2 (0.4)	0.006	94	3.0 (0.7)	2.9 (0.5)	0.436	0.1 (0.0, 0.2)	0.036
Barriers	112	1.5 (1.6)	1.0 (1.4)	0.008	100	1.5 (1.5)	1.4 (1.6)	0.505	−0.1 (−0.3, 0.2)	0.539
Social interaction	113	2.1 (0.7)	2.2 (0.8)	0.253	99	2.1 (0.8)	2.0 (0.8)	0.223	0.1 (−0.1, 0.2)	0.245
Nutrition										
Portion control	104	2.9 (0.8)	3.1 (0.6)	0.081	87	2.7 (0.8)	2.9 (0.8)	0.274	0.0 (−0.1, 0.2)	0.468
Barriers	91	4.5 (2.4)	3.9 (2.2)	0.078	85	4.7 (1.9)	3.6 (2.0)	<0.001	0.3 (−0.1, 0.7)	0.198
Self-efficacy	112	5.2 (1.3)	5.5 (1.0)	0.019	96	4.9 (1.3)	5.4 (1.1)	0.008	0.0 (−0.2, 0.2)	0.974
Diabetes knowledge										
Scale 1	114	5.4 (1.5)	6.4 (1.0)	<0.001	100	5.5 (1.4)	5.9 (1.3)	0.066	0.4 (0.1, 0.6)	0.001
Scale 2	115	3.8 (1.3)	4.6 (1.0)	<0.001	100	4.0 (1.2)	3.6 (1.4)	0.020	0.6 (0.4, 0.8)	<0.001
Physiological measures										
Weight, lbs	114	142.8 (21.6)	140.9 (20.9)	0.014	99	140.6 (22.0)	139.7 (22.6)	0.078	−0.4 (−1.3, 0.5)	0.364
BMI, kg/me	114	25.4 (3.8)	25.1 (3.5)	0.019	99	24.6 (3.1)	24.5 (3.1)	0.489	−0.1 (−0.4, 0.1)	0.205
SBP, mm Hg	113	132.4 (15.7)	130.8 (15.5)	0.194	97	132.5 (18.9)	131.3 (13.8)	0.349	−0.8 (−2.6, 1.0)	0.409
DBP, mm Hg	113	81.8 (11.3)	79.6 (7.6)	0.044	97	82.0 (10.9)	80.7 (8.7)	0.163	−1.0 (−2.3, 0.3)	0.128
Glucose	114	108.3 (23.1)	109.4 (21.5)	0.686	98	112.0 (37.8)	109.3 (20.2)	0.494	1.4 (−2.8, 5.5)	0.528
Cholesterol	113	196.6 (48.9)	192.5 (38.3)	0.394	99	202.4 (57.9)	195.4 (39.3)	0.212	0.0 (−6.6, 6.6)	0.997

OR, odds ratio.

^a^ Adjusted for age, sex, education, years in United States, insurance.

GEE models present the difference in slope both within and between the study groups over time. Greater improvement in moderate and vigorous weekly PA was seen in the treatment group compared with the control group; the different in slopes was 48.1 (95% confidence interval [CI]=4.1, 92.0, *p*=0.032) and 41.2 (CI=20.3, 62.0, *p*<0.001), respectively, in adjusted analyses. Recommended weekly PA at 6 months was seen among a greater percentage of individuals in the treatment group (62.3%) compared with the control group (47.9%). The odds of recommended weekly PA for the treatment group was 1.5 times the odds of recommended weekly PA for the control group in adjusted analysis (95% CI=1.0, 2.1, *p*=0.048).

Greater improvement in PA self-efficacy was also seen in the treatment group compared with the control group; the difference in slopes was 0.1 (95% CI=0.0, 0.2, *p*=0.036). Greater improvement in diabetes knowledge (scale 1 and scale 2) was also seen in the treatment group compared with the control group; the difference in slopes was 0.4 (95% CI=0.1, 0.7, *p*=0.001) and 0.6 (95% CI=0.4, 0.8, *p*<0.001), respectively. Significant group differences were not seen for the other measures in adjusted models (Table 3).

Satisfaction with the intervention was assessed during the 6-month follow-up survey. On a scale of 1–10, 89% reported that they were at least very satisfied (8 or higher). All participants agreed that the program provided at least some education and training on specific strategies to meet diabetes prevention goals, 94% agreed that the program provided at least some assistance to increase personal motivation and confidence, and 86% agreed that the program provided at least some social and peer support.

## Discussion

To our knowledge, this is the first RCT specific to diabetes prevention among Korean American immigrants at risk for diabetes, aside from the pilot study.^37^ This study adds to a limited body of evidence regarding cultural adaptations of the DPP for Asian American subgroups.^46,50,51^

Treatment group participants reported several significant, positive changes between baseline and follow-up, including vigorous, moderate, and recommended weekly PA, PA self-efficacy and barriers to PA, nutrition self-efficacy, and diabetes knowledge, as well as weight, BMI, and DBP. The control group reported significant changes for nutrition barriers and nutrition self-efficacy, as well as one diabetes knowledge scale. Significant differences between the treatment and control group were seen for moderate, vigorous, and recommended weekly PA, as well as PA self-efficacy and diabetes knowledge. Results suggest that a culturally adapted diabetes prevention intervention could be effective in improving behaviors associated with CVD outcomes.

The study sample had relatively low BMIs at baseline (25.1 kg/m^2^), yet significant changes in weight were observed during the intervention period for the treatment group (a mean decrease of 2 lbs). Previous research has supported the use of an Asian-specific BMI threshold of 23 kg/m^2^ (overweight) and 27.5 kg/m^2^ (obese), especially when identifying individuals at risk for diabetes^52,53^; this should be further examined among Korean Americans.

Positive changes were observed for PA outcomes, whereas less significant changes were observed for nutrition outcomes, suggesting that increased PA may have helped increase weight loss among the treatment group. Qualitative findings from the pilot study indicated that treatment group participants were receptive to sessions because they could be applied to their daily lives, and that they were incorporating more walking in daily routines.^37^

It is important to note that the majority of our study participants were older adults. This is particularly important given that the U.S. adult aging population is becoming increasingly diverse in race/ethnicity, language, and culture.^54^ Key strategies for improving older adults' health-related quality of life and healthy aging—physical, mental, and social well-being and functioning^55^—involve supporting lifestyle behaviors such as PA, social engagement, and diet.^56,57^ Racial/ethnic minority older adults may encounter health challenges such as accessing quality health information and services owing to linguistic, cultural, and socioeconomic barriers^58^; therefore, tailored and effective strategies should be developed.^59,60^

Key strategies for improving older adults' healthy aging^55^ involve supporting lifestyle behaviors such as PA,^56,57^ which has the potential to enhance the physical and mental well-being of minority older adults at low cost by strengthening social support networks and leveraging existing community and neighborhood assets.^61,62^ Given that our findings demonstrated enhanced self-efficacy and PA engagement self-efficacy among our study participants, this program may provide a model for engaging older Korean American adults in PA-enhancing strategies.

Several limitations should be mentioned. First, PA and nutrition data were self-reported by participants and thus subject to bias. PA activity was categorized as moderate and vigorous per PA guidelines, with no option for light activities. Participants used checkboxes to pick the type of PA performed, but these activity totals were combined into moderate and vigorous on the survey. Moderate PA may be inflated and should be interpreted with caution. Second, there was potential for cross-contamination, as participants were selected within a community setting where individuals could potentially share information across groups. Third, participants were followed for 6 months, thus we cannot assess the efficacy of the intervention on long-term outcomes.

Fourth, study attrition was high (25% among treatment group participants and 33% among control group participants). Fifth, we did not collect fidelity data, therefore we are unable to confirm the reliability across the CHWs. Sixth, it is not known which participants fasted before the finger pricks were performed; thus, glucose should be interpreted with caution. Finally, our study findings may not be generalizable to other Asian subgroups and settings. However, as our curriculum was adapted from other national programs, we anticipate that the model can be replicated in other communities. In addition, the intervention was linguistically and culturally adapted, and piloted using an iterative process and guided by principles of CBPR for the Korean American population.

## Conclusions

In summary, our findings indicate that a lifestyle-focused community-based diabetes prevention intervention can be effective in promoting PA and healthy lifestyles in an urban, Korean American immigrant community. There is a need to replicate this study with larger samples and in different geographies to assess the generalizability and acceptability of this program and to address the increasing diabetes disparity in this underserved population.

## Abbreviations Used

BMI: body mass index
BP: blood pressure
CBPR: community-based participatory research
CDC: Centers for Disease Control and Prevention
CHW: community health worker
CVD: cardiovascular disease
DBP: diastolic blood pressure
DPP: Diabetes Prevention Program
GEE: generalized estimated equations
NYC: New York City
OR: odds ratio
PA: physical activity
RCT: randomized controlled trial
RICE: Reaching Immigrants through Community Empowerment
SBP: systolic blood pressure
SD: standard deviation
